# Grain Storage: Theory, Technology and Equipment

**DOI:** 10.3390/foods12203792

**Published:** 2023-10-16

**Authors:** Yan Zhao, Haoxin Lv, Yanfei Li

**Affiliations:** School of Food and Strategic Reserves, Henan University of Technology, Zhengzhou 450001, China; lvhaoxin0129@126.com (H.L.); liyanfei@haut.edu.cn (Y.L.)

## 1. Introduction

Grain is the basic material that guarantees human survival, and its security is inextricably linked to the sustainable development and future destiny of humanity. Firstly, this crop provides the body with the energy and nutrients necessary to maintain normal physiological functions. It is rich in carbohydrates, proteins, fats, vitamins, minerals, and dietary fiber, which are essential elements for ensuring human health and survival. Secondly, the abundant consumption of grain provides energy, which can support people’s daily work and provide impetus for the development of human civilization. Thirdly, the stable operation of agricultural production and the grain supply chain is crucial for social stability and economic development.

The “Cereal Supply and Demand Brief” released by the Food and Agriculture Organization of the United Nations on 8 September 2023 predicts that world grain production in 2023 will reach 2.815 billion tons, an increase of 0.9% compared to last year. Although this figure has been rising year by year, the latest report, “2023 The State of Food Security and Nutrition in the World”, points out that there was no progress in addressing global food insecurity in 2022, when an estimated 29.6% of the world’s population were moderately or severely food-insecure. The total number of such individuals has reached 2.4 billion, an increase of 391 million compared to before the COVID-19 outbreak in 2019 [1]. In addition, under the combined influence of various factors such as conflicts, climate change, and economic contraction, the complexity, fragility, uncertainty, and volatility of the international grain supply chain have significantly increased. The stability and resilience of global agricultural and grain industry chains are facing severe pressure. The current international situation emphasizes the importance of grain storage, and advancing the technology involved is vital to ensure a stable quantity of good-quality stored grain.

## 2. Storage Environment and Grain Quality

Rice, wheat, and maize are the most produced and consumed staple cereal crops around the world. Rice and wheat are grown mainly in developing countries in Asia, whereas maize is grown in developing countries of Africa, Asia, as well as South and Central America. In most areas of the world, food crops are seasonally produced and continuously consumed throughout the year. However, due to the limited agricultural mechanization, it has been estimated that post-harvest quantitative loss during storage reaches 15–25% [2]. Stored grains are easily damaged and consumed by insects such as the lesser grain borer (*Rhyzopertha dominica*), maize weevil (*Sitophilus zeamais*), and red flour beetle (*Tribolium castaneum*) and harmful fungi such as *Aspergillus* spp., *Penicillium* spp., and *Fusarium* spp. [3]. The suitable temperature, humidity, and gas composition of grain storage environments will cause the growth and reproduction of pests and fungi. Kaita et al. adopted cold storage (5 °C) to preserve rice grains and achieved good storage efficacy for a storage period of up to 24 months [4]. Lü et al. found that heat treatment could effectively control the infection of *Tribolium castaneum* adults in grain piles during the storage process [5]. Bilhalva et al. reported that integrated monitoring of the equilibrium moisture content and carbon dioxide concentration in a stored corn mass could inform decision making on ideal storage time, loss reduction, and food safety [6]. Qu et al. monitored quality changes in rice during nitrogen-modified atmosphere packaging storage (N_2_-MAPS), with the results showing that this process could delay rice deterioration [7]. Changes in storage environments have a significant impact on grain quality. In order to maintain a high quality during the storage process, effective technologies should be adopted to create a stable environment with the characteristics of low temperature, low moisture, and low oxygen content.

## 3. The Application of Green Grain Storage Technology

Due to the significant impact of changes in storage environment on grain quality, grain depots adopt different technologies to regulate conditions with the aim of effectively reducing loss during storage, ensuring that the grain is free from pests and mold, stays fresh, and has good flavor. Based on consumers’ pursuit of a healthy diet and the importance of ecological protection in various countries around the world, green grain storage is highly popular worldwide; this technology does not use synthetic chemicals and is typified by low-temperature and controlled-atmosphere grain storage technologies.

Low-temperature grain storage technology mainly uses natural or artificial cooling during the storage process to keep grain in the depot at a lower temperature, preventing or slowing down the invasion of harmful organisms and the deterioration in quality. Based on the climate characteristics of grain storage locations, methods such as renovating the thermal insulation of warehouses, closed and covered grain, and natural or mechanical refrigeration cooling are often used to reduce the temperature of grain piles. Ning et al. [8] investigated the quality characteristics of rough rice under low-temperature warehouse conditions using ambient cold air in winter. Four hundred tons of rough rice was stored in the warehouse and aerated from top to bottom using ambient cold air in February. The temperature was maintained below 15 °C without cooling operations until the end of May, and the quality of the rough rice was evaluated in October. The results indicated that compared to ordinary-temperature storage, this method of using ambient winter air better maintained the germination and quality of the rice, induced less change to physiological activity, and resulted in fewer cracked kernels.

Controlled-atmosphere grain storage technology involves manually adjusting the air composition inside the grain storage warehouse to prevent harmful organisms’ metabolic activity, which controls pests, inhibits mold reproduction, reduces the intensity of grain respiration and physiological metabolism, and delays the deterioration in quality. Currently, N_2_ controlled-atmosphere storage is the most widely used method worldwide, using high-concentration nitrogen to replace air in grain piles. Therefore, grain depots implementing this method generally need to purchase nitrogen production equipment, such as carbon molecular sieve and membrane separation nitrogen generators, and require airtightness modifications to the warehouse to maintain a high concentration of nitrogen in the environment. Lorenzo et al. [9] found that when stored corn and wheat grains were exposed to a highly purified N_2_ controlled atmosphere (98.5% ± 0.5), the growth and sporulation of *Fusarium graminearum*, *Fusarium langsethiae*, *Aspergillus flavus*, and *Fusarium verticillioides* and *Aspergillus* aflatoxin production were significantly reduced.

## 4. Green Grain Storage Equipment

Based on the theory of a grain storage ecosystem, the operation mode of temperature- and air-controlled storage, which apply a combination of controlled-atmosphere and low-temperature storage technology, has become the development direction of the field of green grain storage. In a green controlled atmosphere, modern electronic technology and nitrogen supply equipment are harnessed to regulate the supply of nitrogen and sift the air in grain bins through molecular sieves, increasing the nitrogen concentration [10]. The molecular sieves form an air loop with the grain bin, and the nitrogen concentration in the air in the bin remains above 98%. Carbon dioxide gas-controlled grain storage systems include a carbon dioxide gas supply system, a detection system, gas circulation facilities, pressure regulation equipment, an oxygen respirator, a grain monitoring and control system, and a mechanical ventilation system. In addition, during the grain storage process, grain-cooling machines or specialized air conditioners are used to keep the grain in a lower-temperature environment in order to prevent or slow down the invasion of harmful organisms and the deterioration in quality [11]. Internal loop flow temperature control technology accumulates cold sources in the grain pile in winter, and utilizes the supporting circulation equipment installed in the warehouse during the high-temperature season to circulate and equalize grain’s temperature through the operation of the warehouse pipeline, grain pile, and ventilation cage. Therefore, utilizing green, efficient, safe, and energy-saving technologies, continuously exploring the application of new equipment, and ensuring good grain and oil quality represent the development direction of green grain storage technology.

## 5. Perspectives of Grain Storage

Today, due to the rapid growth of the global population and the occurrence of intermittent and unexpected events, grain security is facing significant challenges. It is crucial that more effective technologies and equipment are adopted to improve grain storage efficiency, including minimizing the loss of quantity during the process and effectively maintaining grain freshness. In addition, more attention should be paid to the application of “green grain storage technology” to protect the global ecological environment.

This Special Issue aims to publish high-quality articles on grain storage, covering a wide range of aspects including basic theory, the application of grain storage technology and its impact on quality, and the development of new equipment for grain storage.
